# Compensating for Language Deficits in Amnesia I: H.M.’s Spared Retrieval Categories

**DOI:** 10.3390/brainsci3010262

**Published:** 2013-03-14

**Authors:** Donald G. MacKay, Laura W. Johnson, Vedad Fazel, Lori E. James

**Affiliations:** 1 Psychology Department, University of California, Los Angeles, CA 90095, USA; E-Mail: laurajohnson@ucla.edu; 2 Psychology Department, University of Colorado, Colorado Springs; CO 80918, USA; E-Mail: ljames@uccs.edu

**Keywords:** amnesic H.M., category-specific lexical retrieval, sentence production, propositional conjunction, compensation strategies in amnesia, syntax-based free association, hippocampus, medial temporal lobe

## Abstract

Three studies examined amnesic H.M.’s use of words, phrases, and propositions on the Test of Language Competence (TLC). In Study 1, H.M. used 19 lexical categories (e.g., common nouns, verbs) and one syntactic category (noun phrases) with the same relative frequency as memory-normal controls, he used no lexical or syntactic category with less-than-normal frequency, and he used proper names (e.g., *Melanie*) and coordinative conjunctions (e.g., *and*) with reliably *greater-than-normal* frequency. In Study 2, H.M. overused proper names relative to controls when answering episodic memory questions about childhood experiences in speech and writing, replicating and extending Study 1 results for proper names. Based on detailed analyses of the use (and misuse) of coordinating conjunctions on the TLC, Study 3 developed a syntax-level “compensation hypothesis” for explaining why H.M. overused coordinating conjunctions relative to controls in Study 1. Present results suggested that (a) frontal mechanisms for retrieving word-, phrase-, and propositional-categories are intact in H.M., unlike in category-specific aphasia, (b) using his intact retrieval mechanisms, H.M. has developed a never-previously-observed proposition-level free association strategy to compensate for the hippocampal region damage that has impaired his mechanisms for encoding novel linguistic structures, and (c) H.M.’s overuse of proper names warrants further research.

## 1. Introduction

For many years, researchers assumed that the speech production of amnesic H.M. was intact (see e.g., [1]). However, closer inspection in recent studies indicated abnormalities in how H.M. responded to conversational questions. An example is (1), an excerpt from a conversation with Marslen-Wilson [2] about a lay teacher who took over one of H.M.’s classes in a Catholic grade school:

(1). H.M.: (in response to the question: Why did the lay teacher take over the class?) “Uh … so that they took … well ... she … I say took over, and what I mean it as ... that, as the kids progressed then they were able to … uh … they’d gone to a lay teacher … and they’d seen the nuns around, so when they moved to the grade, next grade, they would … they would naturally … uh … more eased ... with being with the ... uh … nuns than being scared … they were going in there as young kids, they’d be scared, right off in a way ... but they see them around and understand them more”.

H.M.’s response in (1) seems to suggest that the lay teacher took over his class because the pupils would be less fearful with a lay teacher rather than a nun leading the class. However, seeing nuns around, the pupils would become accustomed to them, so that rather than remaining scared, they would feel more at ease when a nun led their next class.

If this response description is accurate, numerous uncorrected errors in (1) obscured H.M.’s intended meaning. For example, in “they would naturally … uh … more eased ... with being with the … uh ... nuns than being scared”, H.M. omitted the verb (*be* or *feel* in *would naturally feel more eased*), he omitted a coordinating conjunction (presumably *rather* in *rather than being scared*), and he substituted a neologism “more eased” for *more at ease* or *more accustomed.* Then, in “what I mean it as that...”, H.M. substituted first “it” and then “as” for *is*, phonological errors that distorted his intended output: *what I mean is that*.

Three aspects of H.M.’s uncorrected errors in (1) are noteworthy. First, such errors are representative rather than exceptional aspects of H.M.’s speech: In well controlled experiments, H.M. has produced: (a) reliably more uncorrected word and phrase omissions than memory-normal controls, as in examples (2)–(4) from MacKay, James, Hadley, and Fogler [3]; (b) reliably more sequencing errors (transpositions, anticipations, and preservations of words and phrases) than memory-normal controls [3], and (c) reliably more neologisms in word reading [4], naming objects on the Boston Naming Test [5] and other tasks [6].

(2). H.M.: “He’s talking on the to somebody” (omission of the word *phone* in: *He’s talking on the phone to somebody*).
(3). H.M.: “There must be a street in between. Because he’s in his office” (omission of the phrase *those buildings and his building* in: *There must be a street in between those buildings and his building because he’s in his office*).
(4). H.M.: “And you can’t tell exactly what it is she’s saying about him. Picture or what” (omission of *whether because of the* in: *And you can’t tell exactly what it is she’s saying about him*, *whether because of the picture or what*).

Second, category-specific aphasics sometimes produce errors that resemble H.M.’s, an observation that raises two important questions: Do H.M.’s language production deficits relative to controls in Corkin [7], MacKay, Burke *et al.* [6], MacKay and James [4,8], MacKay, James, and Hadley [9], MacKay *et al.* [3], MacKay, James, Taylor, and Marian [10], and MacKay, Stewart, and Burke [11] reflect a type of agrammatism? And does the possible or *incipient* left hemisphere damage suggested in Corkin, Amaral, González, Johnson, and Hyman [12] explain H.M.’s language deficits more parsimoniously than his hippocampal region damage?

Third, because the field has recognized the theoretical significance of speech errors since Lashley [13], why did the many researchers interacting informally with H.M. since then overlook his aphasia-like errors and assume that his language skills were “normal” or even “erudite” (see [14])? 

Because these aphasia-linked questions provided the initial impetus for the present research, we first describe the nature of category-specific aphasia and its implications for the retrieval mechanisms underlying normal everyday word, phrase, and sentence production.

### 1.1. Category-Specific Aphasia: Implications for Word Retrieval Mechanisms

Pure and compound category-specific aphasia suggest that the mechanisms underlying word retrieval are category-specific**.** Agrammatic patients with “pure” category-specific aphasia consistently omit or fail to produce words in some lexical categories but not others, although the spared *versus* impaired lexical categories vary from patient to patient, with some patients producing, e.g., nouns but not verbs, and others producing verbs but not nouns (see [15,16,17,18,19,20,21,22,23,24,25,26,27,28,29,30,31,32,33]).

Examples (5a,b) and (6a–c) illustrate pure category-specific aphasia via transcribed excerpts from three famous aphasics who we will simply label X, Y, and Z. Example (5a) is aphasic X’s ungrammatical description of the well-known cookie theft picture in Goodglass and Kaplan ([34], p. 76), and for comparison, (5b) is a model or error-free description of the same picture. Note that aphasic X produced a main verb (*fall over*) and several nouns (*jar*, *chair*, and *water*), but failed to produce other lexical categories seen in (5b), e.g., pronouns (*she*, *her*), determiners (*a*, *the*), auxiliary verbs (*is* in *is trying* and *is standing*), and prepositions (*in*, *onto*).

(5a). Broca’s Aphasic X ([34], p. 76): “Cookie jar … fall over … chair … water … empty … ov … ov … (Expt.: “overflow?”) Yeah.”
(5b). Model cookie theft description: *A woman is in her kitchen doing dishes. She does not notice the boy and girl behind her nor the water flowing out of the sink onto the floor in front of her. The boy is trying to get cookies from a jar high up on a shelf and give one to the girl. He is standing on a stool that is about to fall over*.

Examples (6a) and (6b) illustrate how aphasics Y and Z retold the familiar fox and crow fable, and (6c) is a model description for comparison. Note that unlike aphasic X, aphasic Y produced determiners (*the*), but did not produce several other lexical categories seen in (6c): present participles (*opening*, *dropping*) and infinitives (*to trick* and *to steal)*. However, unlike aphasics X and Y, aphasic Z produced present participles (*singing*), but failed to produce a main verb, an essential lexical category in grammatical sentences.

(6a). Broca’s aphasic Y ([35], p. 38): Well … well … the same thing is s-smart everything, smart … and the brain, OK.
(6b). Broca’s aphasic Z ([36], p. 64): King … Singing … Singing loud … Meat. Perfect!
(6c). Model fable description: *The fox uses flattery to trick the crow into opening its mouth and dropping its cheese for the fox to steal*.

Of course, category-specific aphasia is seldom pure, and compound category-specific aphasics exhibit sequencing and phonological as well as lexical deficits: Besides omitting specific lexical categories, compound category-specific aphasics typically misorder words, omit and/or misorder phonological units, and produce neologisms or jargon for once familiar words (see, e.g., [37,38,39,40]).

Together, pure and compound category-specific aphasia suggest that category-specific activating mechanisms retrieve the sequence of phrases in sentences, words in phrases, and phonological units in words, and can suffer damage that differs from patient to patient (for detailed language production theories sharing this type of category-specific activating mechanism for retrieving word, phrase and phonological units, see [41], pp. 14–61; [4,6,8,9,10]). Thus, aphasic X produced pronouns, determiners, auxiliary verbs, and prepositions, but no nouns or verbs in (5a), suggesting selective damage involving category-specific retrieval mechanisms for activating nouns and verbs, but not pronouns, determiners, auxiliary verbs, or prepositions. By contrast, aphasic Y produced present participles and infinitives but not determiners in (6a), suggesting selective damage involving category-specific retrieval mechanisms for activating determiners, but not present participles and infinitives. Aphasic Z produced main verbs but not present participles in (6b), suggesting selective damage involving category-specific retrieval mechanisms for activating present participles, but not main verbs.

### 1.2. Speech Error Regularities: Further Evidence for Category-Specific Retrieval

Three well-established speech error phenomena known as the lexical class, syntactic class, and phonological class regularities further support the hypothesis that category-specific mechanisms activate the sequence of phrase, word, and phonological units in normal everyday speech production. Table 1 illustrates these regularities for 10 classical types of speech errors. Under the lexical class regularity, words substituted in error virtually always belong to the same lexical class as the intended word (see, e.g., [41], pp. 44–61). For example, verbs substitute in error for intended verbs and not for common nouns or determiners; prepositions substitute in error for intended prepositions and not for proper names or auxiliary verbs; and adjectives substitute in error for intended adjectives and not for conjunctions or pronouns. An example from Burke and Shafto [42] concretely illustrates this lexical class regularity: The speaker (George Bush) intended to say *Take the guns out of the hands of people*, but instead said, “Take the hands out of the guns of people,” where a noun later in the intended sequence (*hands*) substituted an earlier one (*guns*), and *vice versa*. As this typical example illustrates, the speaker twice retrieved the wrong word from the right category, leaving intact the overall sequence of lexical categories in his sentence plan.

**Table 1:** Classical types of everyday speech errors and sequential class regularities: Definitions and examples.

Error Level and Type	Definition	Examples
Types of phonological sequencing errors		
Phonological transpositions, exchanges, or Spoonerisms	Two speech sounds swap positions in the same or different words in a sentence	*left hemisphere*→“heft lemisphere” *well made*→“*mell wade*”
Phonological anticipations	An upcoming speech sound occurs earlier in a word or sentence	*a reading list*→“a leading list” *paddle tennis*→“taddle tennis”
Phonological perseverations	An earlier speech sound reoccurs later in a word or sentence	*escorting*→“escorking”**
Types of sequencing errors involving words and phrases **		
Word anticipations ^b^	An upcoming word or morpheme replaces an earlier one in a sentence	*ministers in the church*→“churches…” *Are you going to be in town on June 22^nd ^*→“Are you going to be on town…”
Phrase transpositions, exchanges, or Spoonerisms	Two phrases in an intended sentence swap positions	*If you stick around you’ll meet him*→“If you meet him you’ll stick around” *I have to smoke a cigarette with my coffee*→“I have to smoke my coffee with a cigarette”
Types of paradigmatic (non-sequential) errors involving words and phrases **		
Word additions ^b^	An unintended word or morpheme is added in an intended sentence	*is wasting away resources →*“*is wasting away of resources*”** ^a ^ *I regret having to inform →*“*I regret for having to inform*”** ^a^**
Word substitutions	An unintended word or morpheme substitutes an intended word or morpheme	*the native values*→“*the native vowels*” *pay be check*→“*pay by rent*”**
Word-level omissions ^b^	An intended word is omitted in the sentence produced	*as much as a surgeon’s knife*→“*as much a surgeon’s knife*”** ^a^**
Word blends	Two context-appropriate words become “fused” together	*hilarity/hysterics*→“*hilarics*” ^a^ *swish/swizzle*→“*swishle*”** ^a^**
Phrase blends	Two context-appropriate phrases become fused together	*Whoever he is/whatever his name is* →“*Whoever his name is*” *I’m going to mainly point out/talk about →*“*I’m going to mainly point about*”**

Intended utterances are in italics. ^a^ indicates examples from [43]; all other examples are from [44]. ^b^ indicates examples irrelevant to the lexical, syntactic, or phonological class regularity; all other examples obey these sequential class regularities.

Under the syntactic class regularity, one phrase substitutes in error for another in the same syntactic class: Noun phrases virtually always substitute in error for intended noun phrases rather than for, say, verb phrases, and verb phrases virtually always substitute in error for intended verb phrases rather than, say, propositions or prepositional phrases. By way of example (from [44]), *We have **a computer** in **our laboratory*** misproduced as “We have **our laboratory** in **a computer**” obeys the syntactic class regularity because the two interchanged phrases (in bold) belong to the same syntactic class (noun phrase; see Table 1).

Under the phonological class regularity, phonological units virtually always substitute in error for intended phonological units in the same syllabic position: Syllable-initial consonants substitute with intended syllable-initial consonants rather than, say, vowels or syllable-final consonants, and syllable-final consonants substitute with intended syllable-final consonants rather than, say, vowels (see, e.g., [45]). By way of example (from [13]), ***d****ear old **qu**een* misproduced as “**qu**eer old **d**ean” obeys the phonological class regularity because both interchanged consonants (in bold) are syllable-initial (see Table 1).

The “sequential class regularity” (a concept encompassing the lexical, syntactic, and phonological class regularities, together with analogous regularities in everyday actions) represents the most general phenomenon established to date in production studies (see [41], pp. 44–61) and applies to transpositions, anticipations, perseverations, blends, and paradigmatic errors involving phrases, words, and phonological units (see Table 1 for definitions and examples).

The sequential class regularity also carries important theoretical implications. One is that direct associative links between specific phrases, words, or speech sounds cannot explain how we activate or retrieve phrases, words, and speech sounds in proper order when we do, and in improper order when we make sequencing errors (as [13] correctly noted). Another implication is that the activating mechanisms for retrieving phrases, words, and speech sounds (in proper or improper order) must be category-specific. For example, anticipation errors must occur when an intended or pre-planned phrase, word, or speech sound is less “primed” or “readied for activation” (Lashley’s original terms) than an upcoming phrase, word, or speech sound *in the same sequential category* when their shared category-specific activating mechanism is applied. As a result, intended and erroneously anticipated phrases, words, or speech sounds are constrained to belong to the same sequential category (for detailed theoretical accounts of sequential class regularities, see, e.g., [41], pp. 44–61; [46]).

### 1.3. Does H.M. Exhibit Category-Specific Aphasia? *[47]*

Under the category-specific aphasia hypothesis, H.M.’s language production deficits resemble category-specific aphasia (either pure or compound), with impaired retrieval and sequencing of some but not all lexical categories (e.g., nouns), some but not all syntactic categories (e.g., noun phrases), and perhaps also some but not all phonological categories (e.g., syllable-final consonants). The category-specific aphasia hypothesis does not specify which types or how many category-specific activating mechanisms have been damaged *versus* spared in H.M. However, if H.M. more often omits and/or misorders words in some categories relative to memory-normal controls, the activating mechanisms governing those categories must be impaired under the category-specific aphasia hypothesis.

Conversely, if H.M. produces units in some categories with no more omission and/or order errors than memory-normal controls, the corresponding category-specific activating mechanisms must be intact under the category-specific aphasia hypothesis. For example, if H.M. omits and/or misorders nouns no more often than memory-normal controls, then his category-specific mechanism for retrieving nouns must be intact.

Consistent with the category-specific aphasia hypothesis, neuroanatomical and theoretical considerations suggest that H.M.’s speech may exhibit *selective* impairment, reflecting damage to *some but not all* category-specific mechanisms for retrieving words and phrases (as in category-specific aphasia). First, H.M.’s lesion could in principle have impaired many category-specific activating mechanisms because English has eight major lexical categories (nouns, verbs, adjectives, adverbs, pronouns, prepositions, conjunctions, and interjections), each of which has several subcategories (e.g., common *versus* proper nouns). Second, some of H.M.’s category-specific activating mechanisms are probably intact because of the fractional nature of his brain damage (see [12]): Except for the amygdala (which triggers emotional reactions), H.M.’s bilateral lesion did not *completely* destroy the hippocampus or any other hippocampal region structure that could in principle house category-specific activating mechanisms for retrieving words, phrases, and speech sounds.

## 2. Studies 1–3 in Overview

The present research consisted of three studies. Study 1 examined how often H.M. and memory-normal controls used 21 lexical categories and one syntactic category (noun phrases) on the Test of Language Competence (TLC) adapted from Wiig and Secord [48] and administered in MacKay *et al.* [9]. If H.M.’s language production deficits reflect category-specific aphasia, we expected that H.M. would reliably underuse some lexical or syntactic categories relative to the controls.

Study 2 followed up on a curious finding in Study 1: H.M. used proper names (e.g., *David*) reliably *more often* than the TLC controls, but he used no lexical category reliably less often. To determine whether H.M.’s overuse of proper names was specific to speech and/or the TLC, Study 2A (spoken responses) and Study 2B (written responses) compared how often H.M. and carefully matched memory-normal controls used proper names when answering episodic memory questions about early childhood experiences.

Study 3 followed up on another curious finding in Study 1: H.M. reliably overused coordinating conjunctions relative to TLC controls. To understand this result, Study 3 analyzed in detail how H.M. and carefully matched controls used (and misused) coordinating conjunctions on the TLC, with results that suggested a “compensation hypothesis” for explaining H.M.’s overuse of coordinating conjunctions and other structures.

### 2.1. Participants

Participants in Studies 1–3 were H.M. and healthy, memory-normal controls recruited through their places of employment in clerical or physical plant positions. The controls were paid for participating and were carefully matched with H.M. for highest educational degree (high school), native language (English), background (semi-skilled labor), age at time of test, and mean verbal and performance IQ scores. H.M.’s combined verbal and performance IQ was 116 at age 44 and 112 at age 71–72.

H.M.’s 1953 sub-orbital suction surgery destroyed virtually the entire amygdaloid complex and partially destroyed several other hippocampal region structures [49]. Partially intact were the entorhinal cortex, the dentate gyrus, the subicular complex, and the posterior half (approximately) of the hippocampal body (although its functional status was never determined). Completely intact were H.M.’s neocortex (including Brodmann’s areas 44/45), temporal stem, parahippocampal cortex, and ventral perirhinal cortex except for where thin metal suction tubes passed bilaterally through his temporal poles [12].

Later in H.M.’s life (1992–1993), magnetic resonance imaging (MRI) in Corkin *et al*. [12] indicated bilateral cerebellar damage (probably due to long-term dilantin use), but cerebellar involvement in H.M.’s language deficits described here is unlikely because of four types of evidence reviewed in MacKay and Johnson [50]. The same MRI study suggested (without data from same-age memory normal controls) “possible” and at most “minimal” damage to lateral temporal neocortex.

Later still (2002–2005), more sophisticated MRI data discounted Alzheimer-related degeneration relative to four memory-normal controls (unmatched with H.M. for IQ, education, or background) but suggested vascular changes and cortical thinning with unknown relations to behavior [51]. These cortical and vascular changes probably followed the present studies (1999), but could have originated earlier (without detection via the relatively insensitive MRI technology in [12]). Possible causes of these cortical and vascular changes include (a) an interaction between normal aging and H.M.’s 1953 lesion (see [4]); and (b) transneuronal dendritic degeneration triggered by his hippocampal lesion, a common occurrence in older adults (see [52,53]).

### 2.2. Database and Procedures: Studies 1 and 3

Because the database for Studies 1 and 3 was the full transcript of participants’ responses in MacKay *et al*. [9], a brief review of their methods and results is in order. The task, a modified version of the TLC, consisted of one practice and 20 experimental trials. The goal on each trial was to create a single grammatical sentence that accurately described a picture and included two or three target words typed below the picture.

Based on stimulus ratings of 10 judges in a preliminary study, MacKay *et al*. [9] categorized the TLC word-picture stimuli as *familiar versus unfamiliar*. The judges rated as familiar, stimuli depicting commonly encountered situations, and containing target words that participated in familiar clichés for describing the pictures; and they rated as unfamiliar, stimuli depicting relatively novel situations and containing target words not part of familiar clichés for describing the pictures.

For unfamiliar stimuli, H.M. included reliably fewer target words than the controls, and described the pictures reliably less accurately, less grammatically, and less completely than the controls (for the criteria used in classifying descriptions as grammatical *versus* ungrammatical and complete *versus* incomplete, see [9]). The responses to an unfamiliar TLC stimulus in (7a,b) illustrate H.M.’s deficits. A general description of the word-picture stimulus appears in (7), followed by H.M.’s description in (7a) and a typical control participant’s in (7b). Note that H.M. described (7) inaccurately (e.g., there was no “lady” in the picture) and omitted the target word *leg* (see 7a), whereas the control participant accurately described the picture and included both target words (see 7b).

(7). DESCRIPTION OF AN UNFAMILIAR WORD-PICTURE STIMULUS:
  *Scene*: A sheer rock cliff in a forest.
  *Protagonists*: Three men, one climbing up the rock face by hand, one pointing up at the climber and talking to the third man, who is listening.
  *Target words*: *fall*, *leg*
(7a). H.M.: “David wanted him to fall and to see what lady’s using to pull himself up besides his hands.”
(7b). Typical Control: “If I fall and break my leg that’s going, not going to be good.”

However, H.M.’s deficits were *selective*: For familiar word-picture stimuli in MacKay *et al*. [9], H.M. and the controls did not differ in accuracy, completeness, or target word inclusion. Moreover, H.M.’s deficits were *graded* rather than all-or-none: Re-presenting the same picture reduced without completely eliminating H.M.’s deficits, and so did asking him to try again (up to seven times) when he failed to include all of the target words (see the complete transcript of all within-trial utterances of H.M. and the experimenter for each word-picture stimulus in the supplementary materials). H.M.’s initial response (8a) and final response (8b) for the word-picture stimulus described in (8) illustrate this effect of repetition. Note that like the typical controls in (8c,d), H.M. produced all three target words without errors on his final but not initial try.

(8). DESCRIPTION OF A FAMILIAR WORD-PICTURE STIMULUS:
  *Scene*: A sidewalk at a street intersection with a traffic light that reads, “Don’t walk”.
  *Protagonists*: A small boy, age about four years old, his father, and his older brother.
  *Action*: The small boy is holding his father’s hand and listens attentively to what his father is saying (presumably about the “Don’t walk” sign). His older brother looks on.
  *Target words*: *before*, *first*, *across*
(8a). H.M. (initial response): “Before at first you cross across.”
(8b). H.M. (final response): “Before you cross the street you have to look both ways first.”
(8c). Typical Control (sole response): “First they waited before walking across the street.”
(8d). Typical Control (sole response): “And the man is telling the little boy that he must look first before he crosses the street.”

### 2.3. Statistical Conventions: Studies 1–3

All statistical analyses followed three non-arbitrary conventions justified in detail in the supplementary materials: For meaningful statistical comparisons, differences between H.M. and the controls in absolute scores had to equal or exceed 4.0; when the control standard deviation (*SD*) was 0.0, the difference between H.M. and the controls was 6.0 *SD*s (rather than ∞); and only differences between H.M. and the controls in excess of 2.0 *SD*s were considered reliable.

## 3. Study 1: Retrieval of Lexical and Syntactic Categories: H.M. *versus* Controls

Study 1 examined whether H.M. underused one syntactic category (noun phrases) and any of 21 lexical categories relative to memory-normal controls on the modified TLC administered in MacKay *et al*. [9]. We first determined the noun phrases (Study 1B) and lexical categories of each word in the participants’ responses (Study 1A), and compared the use frequency of each for H.M. *versus* the controls. If H.M. suffered from category-specific aphasia, we predicted that he would underuse some lexical or syntactic categories but not others relative to the controls.

### 3.1. Study 1A: Retrieval of Lexical Categories

Study 1A examined how often participants on the TLC used words in 21 lexical categories, including nouns (common and proper), pronouns, verbs (transitive, intransitive, and auxiliary), noun modifiers (indefinite articles, definite articles, canonical adjectives, demonstrative adjectives, and possessive adjectives), verb modifiers (canonical adverbs, time adverbs, and frequency adverbs), prepositions (canonical prepositions, place prepositions, and time prepositions), conjunctions (coordinating, subordinating, and correlative), and interjections.

#### 3.1.1. Method

##### 3.1.1.1. Participants

The participants were H.M. at age 72 and eight controls who did not differ reliably from H.M. in mean age (70; *SD* = 4.6) or mean combined verbal and performance IQ score (113; *SD* = 9.67).

##### 3.1.1.2. Database and Procedures

The database was the full set of transcribed responses of H.M. and the controls on the TLC (see [9] for detailed transcription procedures). The goal on each trial was to accurately describe a picture using two or three pre-specified target words in a single grammatical sentence, and a response was defined as a string of words bounded by trial onset, trial offset, or a substantive comment from the experimenter (e.g., a request to try again). We chose this database as providing more useable data for lexical category analyses than the smaller MacKay *et al.* [9] database, which included only H.M.’s best response on each TLC trial. H.M.’s responses in the present database are shown in the supplementary materials, together with a model (complete and error-free) description for the practice and experimental stimuli.

To prepare the database for lexical category analyses, we edited out irrelevant aspects of the responses, including self-corrected errors and error markers (e.g., “no”, “I mean”, “sorry”, “um”, “er”, and “not”), experimenter comments (e.g., “OK”, “good”, and “mm hm”), on-line revisions or repetitions (e.g., “bus ... school bus”), interjections and other common dysfluencies (e.g., “um” and “uh”), word and phrase repetitions, false starts, and extraneous or off topic comments (e.g., “it isn’t pointed out here what it is”, and “no that doesn’t work”). These edited-out aspects became part of the speech error analyses in MacKay, Johnson and Hadley [54].

As main analyses, we tabulated the lexical category of each word in the database using the sentence context together with the lexical class specifications in Dictionary.com. We then computed the use frequency for common nouns (e.g., *enemy*, *uncle*, *goal*), proper names (e.g., *Canada*, *Sandy*), transitive verbs (e.g., *toss*, *love*), intransitive verbs (e.g., *exist*, *stink*), auxiliary verbs (e.g., *could*, *should*), canonical adjectives (e.g., *diligent*, *red*, *short*), demonstrative adjectives (e.g., *this*, *those*), possessive adjectives (e.g., *your*, *her*), adverbs of time (e.g., *yesterday*, *soon*), adverbs of frequency (e.g., *often*, *sometimes*), pronouns (e.g., *she*, *we*, *his*, *yours*, *himself*), canonical prepositions (e.g., *of*, *for*), prepositions of time (e.g., ***at*** *6:00*, ***for** a year*), prepositions of place (e.g., ***at*** *my place*, ***in** the box*), coordinating conjunctions (e.g., *and*, *or*, *but*), subordinating conjunctions (e.g., *although*, *after*, *because*), indefinite articles (e.g., *a/an*), and definite articles (e.g., *the*).

#### 3.1.2. Results and Discussion

##### 3.1.2.1. Absolute Use Frequencies

Table 2 shows absolute use frequencies of lexical categories in the full set of transcribed responses for H.M. and the controls (means and *SD*s). H.M. and the controls both used 19 of the lexical categories, but the controls used correlative conjunctions (e.g., *either*/*or* and *both*/*and)*, whereas H.M. did not, and H.M. used proper names (e.g., *Melanie*, *David*,and*Gary)*, whereas the controls did not (see Table 2)*.* However, H.M. used 955 words overall *versus* a mean of 233 (*SD* = 120.88) for the controls, a reliable 5.98 *SD* difference that rendered absolute use frequencies unsuitable for statistical analysis and called for the analyses of relative use frequency examined next.

**Table 2:** Absolute and relative use frequency of lexical categories for all words in Study 1.

General Lexical Category	Specific Lexical Category	Examples	Absolute Use Frequency		Relative Use Frequency			
			H.M.	Controls	H.M.	Controls Mean	Controls *SD*	Frequency Difference Scores in *SD*s
Nouns	Common Nouns	*enemy*, *uncle*, *goal*	108	33.40	11.31	14.20	3.21	−0.90
	Proper Nouns	*Canada*, *Sandy*	7	0.00	0.73	0	0	6.00 *
Pronouns	Pronouns	*she*, *we*, *his*, *yours*	146	28.40	15.29	11.98	3.32	1.00
Nominal Modifiers	Indefinite Articles	*a*/*an*	7	4.80	0.73	1.99	1.62	−0.78 ^a^
	Definite Articles	*the*	26	12.80	2.72	5.78	4.69	−0.65
	Canonical Adjectives	*diligent*, *red*, *short*	61	17.20	6.39	8.50	2.91	−0.73
	Demonstrative Adjectives	*this*, *those*	37	6.80	3.87	2.82	0.98	1.07
	Possessive Adjectives	*your*, *her*	9	3.00	0.94	1.20	0.74	−0.35
Verbs	Main Verbs: Transitive	*toss*, *love*	101	19.60	10.58	9.18	2.20	0.64
	Main Verbs: Intransitive	*exist*, *stink*	125	27.60	13.06	11.87	1.98	0.62
	Auxiliary Verbs	*could*, *should*	65	24.20	6.81	9.82	2.68	−1.12
Verb Modifiers	Canonical Adverbs	*really*, *not*, *only*	98	19	10.26	7.81	2.69	0.91
	Adverbs of Time	*yesterday*, *soon*	5	1.00	0.52	0.66	0.64	−0.21
	Adverbs of Frequency	*often*, *sometimes*	1	0.20	0.10	0.10	0.21	0.04 ^a^
Prepositions	Canonical Prepositions	*of*, *with*	17	6.8	1.78	2.55	1.30	−0.59
	Prepositions of Time	*at* 6:00, *for* a year	3	0.60	0.31	0.42	0.71	−0.15 ^a^
	Prepositions of Place	*at* my place, *in* the box	25	5.20	2.62	1.83	1.16	0.68
Conjunctions	Coordinating Conjunctions	*and*, *or*, *but*	68	9.60	7.12	3.92	0.93	3.45 *
	Subordinating Conjunctions	*although*, *after*, *because*	32	9.00	3.35	3.46	1.33	−0.08
	Correlative Conjunctions	*either*/*or*, *both*/*and*	0	1.60	0.00	1.09	1.42	−0.77 ^a^
Interjections	Interjections	*well*, *oh*	14	1.8	1.47	0.84	0.59	1.06
*N*/%			955	232.60	99.96	100.02		

Relative frequency difference scores are the relative use frequency for H.M. minus the mean for controls (in *SD*s). * indicates a statistically reliable difference score; ^a^ indicates differences in absolute *N*s too small for meaningful statistical analysis.

##### 3.1.2.2. Relative Use Frequencies

Table 2 shows relative frequencies by lexical category for H.M. and the controls (means and *SD*s), with relative use frequency calculated as the absolute use frequency for a lexical category divided by overall size of a participant’s edited transcript multiplied by 100. Also shown in Table 2 are the relative frequency difference scores, calculated as the relative use frequency for H.M. minus the mean for the controls divided by the control *SD* for each lexical category*.* Relative frequency difference scores ranged from −1.12 to +6.0 *SD*s but were never meaningfully greater for the memory-normal controls than H.M. for any lexical category: Although the controls used relatively more correlative conjunctions than H.M., the difference in absolute *N* for H.M. (0) *versus* the control mean (1.60) was too small for meaningful analysis. Absolute *N*s were likewise too small for meaningful analyses of relative use frequencies for indefinite articles, prepositions of time, and adverbs of frequency (see Table 2).

However, relative use frequencies for two lexical categories were reliably greater for H.M. than the controls: coordinating conjunctions (a 3.45 *SD* difference) and proper names (a 6.0 *SD* difference by convention), findings reminiscent of H.M.’s overuse of cliché phrases in MacKay, Burke *et al.* [6]. To rule out H.M.’s reduced target word use as a factor in these results, we reanalyzed the database excluding the target words, and relative use frequency in this second analysis was again reliably greater for H.M. than the controls for both proper names (6.0 *SD*s by convention) and coordinating conjunctions (2.07 SDs; see Table 3).

**Table 3:** Absolute and relative use frequency of Study 1 lexical categories, excluding target words.

General Lexical Category	Specific Lexical Category	Examples	Absolute Use Frequency		Relative Use Frequency				
			H.M.	Controls	H.M.	Controls Mean	Controls *SD*		Frequency Difference Scores in *SD*s
Nouns	Common Nouns	*enemy*, *uncle*, *goal*	81	28.60	9.44	14.14	3.56		−1.32
	Proper Nouns	*Canada*, *Sandy*	7	0.00	0.82	0.00	0		6.00 *
Pronouns	Pronouns	*she*, *we*, *his*, *yours*	146	27.6	17.02	13.97	4.58		0.66
Nominal Modifiers	Indefinite Articles	*a*/*an*	7	4.80	0.82	2.43	2.12		−0.76
	Definite Articles	*the*	26	12.80	3.03	6.77	5.36		−0.69
	Canonical Adjectives	*diligent*, *red*, *short*	44	10.20	5.13	5.63	1.91		−0.23
	Demonstrative Adjectives	*this*, *those*	37	6.80	4.31	3.39	1.26		0.73
	Possessive Adjectives	*your*, *her*	9	3.00	1.05	1.40	0.90		−0.39
Verbs	Main Verbs: Transitive	*toss*, *love*	95	17.60	11.07	9.40	1.78		0.94
	Main Verbs: Intransitive	*exist*, *stink*	111	21.80	12.94	10.78	2.15		1.01
	Auxiliary Verbs	*could*, *should*	65	23.20	7.58	11.19	2.92		−1.24
Verb Modifiers	Canonical Adverbs	*really*, *not*, *only*	88	18.40	10.26	9.01	3.43		0.36
	Adverbs of Time	*yesterday*, *soon*	2	0.20	0.23	0.07	0.16		^a^
	Adverbs of Frequency	*often*, *sometimes*	1	0.20	0.12	0.11	0.24		^a^
Prepositions	Canonical Prepositions	*of*, *with*	17	6.60	1.98	2.91	1.63		−0.57
	Prepositions of Time	*at* 6:00, *for* a year	1	0	0.12	0.00	0.00		^a^
	Prepositions of Place	*at* my place, *in* the box	24	4.80	2.80	1.96	1.15		0.72
Conjunctions	Coordinating Conjunctions	*and*, *or*, *but*	58	7.40	6.76	2.92		1.85	2.07 *
	Subordinating Conjunctions	*although*, *after*, *because*	25	5.60	2.91	2.19		1.63	0.45
	Correlative Conjunctions	*either*/*or*, *both*/*and*	0	0.80	0.00	0.70		0.91	^a^
Interjections	Interjections	*well*, *oh*	14	1.8	1.63	1.03		0.75	0.80
*N*/%			858	202.20	100.02	100.00			

* indicates a statistically reliable difference score; ^a^ indicates absolute *N*s too small for meaningful statistical analysis.

In summary, the controls exhibited reliably greater relative use frequency than H.M. for no lexical category, contrary to the category-specific aphasia hypothesis, and neither sample size nor target word inclusion constrained this conclusion. However, three puzzling results in Study 1 warranted further research: H.M.’s reliable overuse of proper names and coordinating conjunctions relative to the controls, and his non-use of correlative conjunctions, e.g., *either*/*or*, and *both*/*and* (an ambiguous result because differences in absolute *N*s for H.M. *versus* the controls for this lexical category were too small for meaningful analysis).

Understanding these puzzling results was a primary goal in Studies 2–3 and MacKay *et al.* [54]. Study 2 examined whether H.M. overused proper names in new tasks administered at a younger age, and MacKay *et al.* analyzed H.M.’s use and misuse of proper names and correlative conjunctions in detail. Study 3 took parallel steps to understand H.M.’s Study 1 overuse of coordinating conjunctions by analyzing his use and misuse of coordinating conjunctions in detail.

### 3.2. Study 1B: Retrieval Frequency of Noun Phrases: H.M. *versus* the Controls

Study 1B resembled Study 1A except that a syntactic structure was the unit of analysis. By definition, syntactic structures combine one or more words to form a phrase or proposition, and Study 1B analyzed how often H.M. and the controls retrieved noun phrases, a major syntactic structure in English. In standard definitions [55,56,57,58], noun phrases combine a noun with modifiers or complements, as in *that important point*, a noun phrase with head noun *point* and two modifiers: a demonstrative adjective * (that)* and a canonical adjective (*important*).

The question in Study 1B was whether H.M. uses noun phrases with lower relative frequency than memory-normal controls, as in a subclass of category-specific aphasia where the ability to construct or retrieve some syntactic structures but not others is impaired. By way of illustration, aphasic Y in (6a) used noun phrases (e.g., *the same thing*, and *the brain*) and verb phrases (e.g., *is smart*) but not the complement structures expected in normal descriptions of the fox and crow fable, e.g., *for the fox to steal* and *to trick the crow* in (6c), which suggests impairment in constructing or retrieving complement structures but not noun phrases or verb phrases.

#### 3.2.1. Method

Participants and procedures were identical to Study 1 except that we used the smaller TLC database of MacKay *et al.* ([9]; see the supplementary materials) because it contained only H.M.’s best response on any given TLC trial, thereby reducing the number of uncorrected grammatical errors that could complicate the syntactic structure analyses in Study 1B.

Study 1B tabulated all multi-word noun phrases in this database for H.M. and the controls, ignoring errors (see [54] for detailed error analyses) and single-word noun phrases (because Study 1 had already analyzed single-word usage).

#### 3.2.2. Results and Discussion

The mean number of noun phrases per response was 1.72 for H.M. *versus* 2.21 (*SD* = 1.38) for the controls, a non-reliable 0.36 *SD* difference. The mean number of words per noun phrase also did not differ for H.M. (2.32 words) *versus* the controls (2.21 words; *SD* = 0.55), a non-reliable 0.60 *SD* difference. These results indicate that H.M. did not underuse noun phrases relative to the controls, and suggest that (a) he did not suffer category-specific aphasia involving noun phrases, and (b) his category-specific mechanisms for retrieving noun phrases were intact.

## 4. Study 2: Proper Name Use in Answering Episodic Memory Questions

Study 2 followed up on the reliably greater use of proper names (e.g., *Gary*) for H.M. than memory-normal controls in Study 1. To determine whether this result was specific to the TLC, to spoken speech, or to H.M.’s age (72 in Study 1), Study 2 examined H.M.’s proper name use in spoken and written episodic memory tasks at age 44 and 71. In both tasks, H.M. and age-matched memory-normal controls answered episodic memory questions concerning childhood events, an appropriate domain choice because H.M.’s early childhood memories are intact by common assumption (see e.g., [59]). However, answers were spoken in Study 2A *versus* written in Study 2B.

### 4.1. Study 2A: H.M.’s Spoken Use of Proper Names at Age 44

Study 2A used analytic procedures resembling Study 1A to tabulate use frequencies for an experimental category (proper names) and a control category (pronouns) in transcripts of spoken answers to episodic memory questions concerning childhood events. We chose pronouns as the appropriate control category for proper names because (a) proper names and pronouns represent equivalent ways of designating a referent, e.g., a person or object, and (b) unlike proper names, pronouns did not differ in use frequency for H.M. *versus* the controls in Study 1. Under the assumption that neither task nor age influenced Study 1 results, we expected identical results in Study 2A: reliably greater use for the experimental category (proper names) but not the control category (pronouns) for H.M. relative to the controls.

#### 4.1.1. Method

##### 4.1.1.1. Participants

The participants were H.M. at age 44, and seven controls with mean age 45 and combined verbal and performance IQ 117.72 (*SD* = 13.40), a non-reliable 0.10 *SD* difference relative to H.M.

##### 4.1.1.2. Materials and Procedures

The materials were six episodic memory questions from Marslen-Wilson [2], a 182-page transcript of conversations between Marslen-Wilson and H.M. at age 44. All six questions addressed childhood experiences that occurred prior to age nine, e.g., *What is your first or earliest memory?* Excluded were questions calling for explicit recall of proper names and “follow-up” questions that Marslen-Wilson asked about earlier H.M. responses (thereby ensuring comparable response contexts for H.M. and the controls).

Following Marslen-Wilson’s [2] procedures as closely as possible, the controls heard the questions in one-on-one conversations with an experimenter in a laboratory setting and their spoken responses were tape-recorded and later transcribed (see [9] for transcription procedures). As in Study 1, we then tabulated the use frequency of proper names and pronouns from the transcripts.

#### 4.1.2. Results and Discussion

The mean number of words per response was 617 for H.M. *versus* a mean of 244.86 for the controls (*SD* = 116.19), a reliable 3.20 *SD* difference that called for relative frequency analyses of our main results.

##### 4.1.2.1. Relative Frequency Analyses

Consistent with Study 1 results, proper names made up 6.48% of H.M.’s words *versus* a mean of 2.58% for the controls (*SD* = 1.48%), a reliable 2.64 *SD* difference favoring H.M. Example (9a,b) illustrates this finding for H.M. and a typical control participant responding to the question “What is your first memory?”: The control used no proper name words (see 9b), whereas H.M. used five: *Hartford*, *Manchester*, *South Coventry*, and *Burnside* (see 9a).

(9). Experimenter question: “What is your first memory, the earliest thing you remember?”
(9a). H.M.: When I ... tell you that ‘tis ... you see ... may have been ... that was when I was going to high school ... that … and ... but before that when I was going to the private kindergarten, two houses up, from where I lived, when I went to high school, but the other places I lived in Hartford, and Manchester, and then South Coventry ... before coming back to (chuckles) Burnside avenue again.
(9b). Typical control participant: “Oh, way back, uh ... two. I was two because I have seen pictures of myself in a snowsuit, and I outgrew it very quickly, but when I was two I wore it and when I was two I remember walking in my grandma’s kitchen and pointing up at my snowsuit hanging on the kitchen door because I wanted to put it on, and it’s very clear—it was light blue.”

Also consistent with Study 1 results, pronouns made up 4.86% of H.M.’s words *versus* a mean of 4.91% for the controls (*SD* = 2.00%), a non-reliable difference.

##### 4.1.2.2. Type and Token Analyses: Pronouns and Proper Names

To illustrate the distinction between types *versus* tokens, H.M. retrieved 10 different types of proper names overall (*Burnside Avenue*, *Connecticut*, *East Hartford*, *Frankie*, *Hartford*, *Jimmie Wood*, *L.T. Wood*, *Manchester*, *South Coventry*, and *Spruce Street*) and the seven controls retrieved 38 different proper name types overall (*Bad Peter*, *Black*, *Carter*, *Camp David*, *Central City*, *Colorado*, *Christmas*, *Denver*, *Drew Bryant*, *Easter*, *F-15 fighter*, *Mrs*. *Folgers*, *Germany*, *Gigantic Cleaners*, *Harley*, *Halloween*, *Hitler*, *Hog Days*, *Illinois*, *JFK*, *Jerry Lewis*, *Kentucky*, *Kewanee*, *Labor Day*, *Ms*. *Hanbee*, *New York*, *Nixon*, *Pokie*, *Puyallup*, *Reagan*, *SALT I*, *SALT II*, *Satan*, *Saturday*, *Susan*, *Tehran*, *Vietnam*, *Westwood Elementary*). However, H.M. retrieved 24 proper name tokens because he repeated *Burnside Avenue* seven times, *East Hartford* six times, and *Hartford* once, and the seven controls retrieved 52 proper name tokens overall because they repeated *Labor Day* and *New York* three times, *Carter* twice, and *Denver*, *Easter*, *Harley*, *Hog Days*, *Jerry Lewis*, and *Reagan* once.

The present type and token analyses used lexical items rather than words as the unit of analysis. To illustrate this distinction, *South Coventry* represents a single name or lexical item but contains two words, so that (9a) contained five proper name words but only four lexical items: *Hartford*, *Manchester*, *Burnside*, and *South Coventry*. After counting the pronoun and proper name types and tokens for each participant, we calculated tokens-per-type ratios as a measure of how often participants repeated units that they used.

H.M. used no more pronoun types than the controls, with 6 different pronoun types for H.M. *versus* a mean of 4.17 for the controls (*SD* = 1.07), a non-reliable 1.71 *SD* difference. The tokens-per-type ratio for pronouns also did not differ for H.M. (5.83) *versus* the mean for the controls (3.85; *SD* = 2.27), a non-reliable 0.87 *SD* difference.

The parallel tokens-per-type analysis for proper names yielded 10 proper name types for H.M. *versus* a mean of 6.33 for the controls (SD = 3.43), a non-reliable 1.07 *SD* difference. However, the tokens-per-type ratio was 2.4 for H.M. *versus* a mean of 1.09 for the controls (*SD* = 0.123), a reliable 10.65 *SD* difference.

We repeated our tokens-per-type analyses for pronouns and proper names using relative frequencies as the unit of analysis and obtained the same results, ruling out the larger number of words in H.M.’s output as a possible explanation for his tendency to repeat proper names. Also ruled out as a factor were the topics of the questions because H.M.’s proper name use was usually irrelevant to Marslen-Wilson’s questions, reflecting a deliberate topic shift to proper names (see, e.g., 9a).

In summary, our tokens-per-type analyses indicated that H.M. repeated proper names but not pronouns reliably more often than memory-normal controls. This finding again indicates that proper names represent a special lexical category for H.M., and calls for qualification of the generalization that H.M. has a general tendency to repeat [3,6,11]. Despite repeating a wide range of forms reliably more often than memory-normal controls, including familiar stories, paragraphs, sentences, phrases, and common clichés, H.M. does not have a *general* tendency to repeat because he repeated proper name types but not pronoun types more often than controls.

##### 4.1.2.3. Content Analyses of Proper Name Use

For participants using four or more proper name types, we analyzed the content of their proper names. These analyses revealed two reliable content differences between H.M.’s proper names *versus* the controls’. First, 70% of H.M.’s proper names were place names (e.g., *Burnside Avenue*, *Connecticut*, *East Hartford*, *Hartford*, *Manchester*, *South Coventry*, and *Spruce Street*) *versus* a mean of 23.3% for the controls (*SD* = 6.11), a reliable 7.64 *SD* difference. Second, 70% of H.M.’s place names were street and city names *versus* a mean of 13% for the controls. Overall then, 49% of H.M.’s proper names were street (e.g., *Burnside Avenue*, *Spruce Street*) and city (e.g., *East Hartford*, *Hartford*, *Manchester*, and *South Coventry*) names *versus* a mean of 3.03% (*SD* = 6.41) for the controls, a reliable 7.17 *SD* difference.

Were H.M.’s street names accurately recalled episodic memories or were they imagined or fabricated? To illustrate this issue, H.M.’s repeated reference to high school in example (9a) represents an unlikely “first childhood memory” because high school by definition falls outside early childhood.

Although appropriate in some contexts, memory-type and memory-accuracy questions are inappropriate in the present context: When comparing the use frequency of equivalent ways to express the same concept, here proper names *versus* pronouns, it matters not whether the basis for use is irrelevant discourse, imagined facts or events, or memories for semantic facts *versus* unique personally experienced events.

### 4.2. Study 2B: H.M.’s Written Use of Proper Names at Age 71

Study 2B resembled Study 2A except that the participants were older and answered visually presented episodic memory questions in writing rather than speech in order to test whether memory factors influenced Study 1 results: With written stimuli and responses, participants needed to recall neither the questions nor their answers (as they unfolded), and we expected different results in Study 2B if these memory factors affected prior results, but the same results (greater proper name use for H.M. than the controls) if they did not affect prior results.

#### 4.2.1. Method

##### 4.2.1.1. Participants

The participants were H.M. at age 71, and three controls with mean age 70 (range 67–74) and combined verbal and performance IQ 119.1 (*SD* = 5.02), a non-reliable 1.41 *SD* difference relative to H.M.

##### 4.2.1.2. Procedures and Materials

The participants received a five-page booklet with an autobiographical question heading each page, followed by the instruction: Write as much as you want in answering the question. It is not necessary to fill the entire page. Do not worry about exact spelling.

The experimenter repeated the instructions and read each question aloud for H.M. but not the controls. The questions were: *What is your earliest memory? Can you describe any children in your kindergarten class? Can you describe any children in your grade school? Describe any single event when you were 7 or younger involving your mother. Describe any single event when you were 7 or younger involving your father*. Response duration was determined via stopwatch.

#### 4.2.2. Results and Discussion

##### 4.2.2.1. Number of Words per Response

We removed from analyses two questions eliciting one-word and irrelevant responses: H.M. and two controls who had not attended kindergarten answered “no” to *Can you describe any children in your kindergarten class?*; and H.M. answered *Can you describe any children in your grade school?* with an irrelevant string of abbreviated proper names (see (10)). In response to experimenter questions following (10), H.M. indicated that “MAN.” stood for *Manchester*; “S.P.S.” for *Saint Peter’s School*; and “HTFD” for *Hartford Fire Department*, and we decided that including this irrelevant response would have biased present results in favor of our hypothesis (greater proper name use for H.M. than the controls).

(10). H.M. (written answer to the question *Can you describe any children in your grade school?* Underlining and punctuation as per the original): MAN. S.P.S. HTFD.

For the remaining questions, the overall mean number of words per response was 17.67 for H.M. *versus* 26.56 for the controls (*SD* = 4.44), a reliable 2.00 *SD* difference that called for relative frequency analyses of our main results.

##### 4.2.2.2. Relative Use Frequency

Proper names made up 11.32% of the words in H.M.’s responses *versus* a mean of 1.24% for the controls’ (*SD* = 2.11), a reliable 4.81 *SD* difference. This replication of earlier results indicated that (a) H.M. retrieved proper names with greater-than-normal frequency when written questions and responses obviated the need to recall either the questions or his own ongoing responses, and (b) H.M. overused proper names in three tasks: answering episodic memory questions about childhood events in speech and writing and creating spoken sentences on the TLC (Study 1).

##### 4.2.2.3. Response Duration

Mean overall response durations were about 248 s for H.M. *versus* 100 s (*SD* = 34) for the controls, a reliable 4.35 *SD* difference attributable in part to H.M.’s cerebellar damage. Because the controls produced reliably more words per response than H.M., mean time per word was also reliably longer for H.M. than the controls.

##### 4.2.2.4. Uncorrected Errors

**Figure 1:**
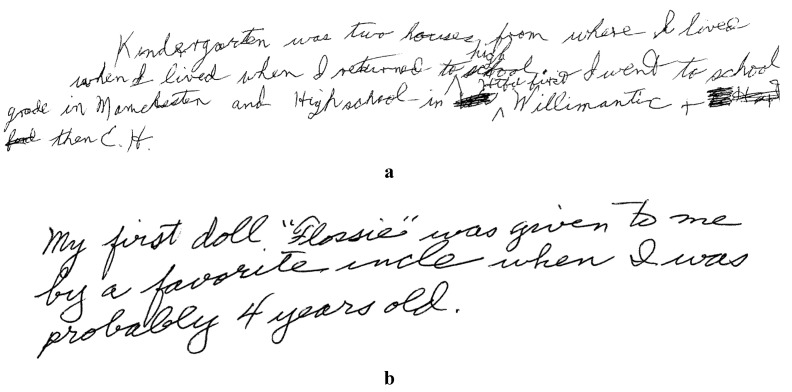
Handwritten responses to the question *What is your earliest memory?* with proper names italicized in a verbatim transcription. (**a**) H.M.: “Kindergarten was two houses from where I lived when I lived when I returned to high school. First I went to school grade in *Manchester* and High school in *Htfd Willimantic* + then *E.H.*” (*Htfd* represents Hartford; *E.H.* represents East Hartford). (**b**) Typical control participant: “My first doll “*Flossie*” was given to me by a favorite uncle when I was probably 4 years old”.

With misspellings excluded, H.M. produced more uncorrected errors than the controls. H.M.’s handwritten response to the question *What is your earliest memory?* illustrates two such errors (see Figure 1): “school grade” instead of *grade school*, and “where I lived when I lived when I returned to high school”, where H.M. presumably failed to cross out *when I lived*, a noteworthy non-correction because (a) this error rendered his sentence ungrammatical, and (b) H.M. crossed out several lesser errors in Figure 1.

Overall, H.M. produced eight uncorrected word- and phrase-level errors *versus* a mean of 0.60 for the controls (*SD* = 0.35), a reliable 21.14 *SD* deficit. This finding extends H.M.’s deficits in correcting self-produced errors to written speech and rules out time constraints and problems in recalling his just-produced output as causal factors: In Study 2B, there were no time constraints and H.M. could see and correct his handwritten responses without having to recall his prior output.

##### 4.2.2.5. Response Coherence

Although coherence or relevance problems were too infrequent for meaningful statistical analyses, H.M. produced several notable examples, such as his reference to *high school* in Figure 1, which was clearly incoherent with the topic, *your earliest memory*. Because H.M. could have maintained coherence by reading the questions and his own written responses in Study 2B, such examples suggestthat forgetting or memory problems cannot fully explain his basically similar coherence problems in MacKay, Burke *et al*. [6], and MacKay *et al*. [3,9,10].

##### 4.2.3. Subsidiary Results: Unusual Abbreviations, Letter Cases, Underlining, and Graphemic Errors

Graphemic characteristics differed for H.M. *versus* the controls in two ways: graphemic fluency and unusual abbreviations, letter cases, and underlining. Handwriting was more fluent and less error-prone for the controls than H.M., a difference attributable to H.M.’s cerebellar damage. For example, in Figure 1, H.M. substituted M for the N in *Manchester*, and retraced the A in *Kindergarten* and the O in *houses*, whereas the controls virtually never retraced or misproduced letters.

Unlike the controls, H.M. also produced unorthodox abbreviations, letter cases, and underlining. For example, H.M. abbreviated *East Hartford* as “E.H.” in Figure 1, perhaps to economize on the effort that his cerebellar motor difficulties demanded. However, motor difficulties cannot explain H.M.’s capitalization errors and unorthodox use of underlining, as when he underlined the pronoun *I* for no apparent reason, incorrectly capitalized the first *H* in *high school*, and failed to capitalize the sentence-initial word *First* in Figure 1, all without correction. By contrast, the controls never produced unusual abbreviations, inappropriate case, or inexplicable underlining, a reliable 6.0 *SD* difference by convention (see the typical control response in Figure 1).

### 4.3. General Discussion

H.M.’s overuse of proper names in Studies 1 and 2 has no simple explanation and warrants further research. For example, H.M. did *not* overuse proper names because they are easily retrieved or encoded: Proper names are in fact more difficult to encode and retrieve than other types of information about people such as their (common noun) occupation (see, e.g., [60,61,62]). However, based on extensive analyses of encoding and retrieval errors on the TLC, MacKay *et al*. [54] concluded that H.M.’s overuse of proper names reflects compensation processes resembling those examined in Study 3.

## 5. Study 3: Compensation Underlying H.M.’s Use and Misuse of *and*

The question in Study 3 was why H.M. used reliably more coordinating conjunctions than memory-normal controls in Study 1. As a first step in addressing this question, we analyzed how often participants used various types of coordinating conjunctions on the TLC. To anticipate the results of these use frequency analyses, H.M. overused *and* but no other coordinating conjunction relative to the controls. This finding called for further analyses of how H.M. used and misused *and*, and results of those analyses suggested that H.M. overused *and* to compensate for deficits in creating novel sentence-level plans.

### 5.1. Method

#### 5.1.1. Participants and Database

The participants and database were identical to Study 1.

#### 5.1.2. Procedures

We first analyzed the use frequency of three types of coordinating conjunctions in the TLC database: *and (* as in *I went to Boston **and** Cambridge)*, *but* (as in *He shot*, ***but** missed)*, and *so* (as in *I stood up **so** I could see*). Because *or*, as in *Did you walk **or** take a cab*, was a target word, we did not analyze use frequency for this fourth type of coordinating conjunction (but see [54] for detailed analyses of H.M.’s use and misuse of *or*). Based on the results of our use frequency analyses, Study 3 then analyzed how H.M. used (and misused) *and*, together with related ways of conjoining propositions (e.g., temporal and causal subordinating conjunctions).

### 5.2. Main Results

#### 5.2.1. Use Frequency of *and*, *but*, and *so*

The mean use frequency of *and* was 0.057 per word for H.M. *versus* 0.024 for the controls (*SD* = 0.016), a reliable 2.06 SD difference, with more instances for H.M. than the controls. The mean use frequency of *but* was 0.003 per word for H.M. *versus* 0.010 for the controls (*SD* = 0.007), a non-reliable 1.0 SD difference. The mean use frequency of *so* was too infrequent for meaningful statistical analysis: 0.001 per word for H.M. *versus* 0.057 per word for the controls (*SD* = 0.016). In short, *and* was the only non-target coordinating conjunction that H.M. used reliably more often than the memory-normal controls.

#### 5.2.2. The Functions of *and*

As a first step toward understanding why H.M. overused *and*, we analyzed use frequencies for the three major functions of *and*: to conjoin individual words (e.g., *Mary **and** I*), to conjoin noun phrases (e.g., *the administration building **and** its inhabitants*, and verb phrases (e.g., *have our cake **and** eat it too*), and to conjoin propositions (e.g., *She wants to behave herself **and** he likes that*). Unlike the controls, H.M. only used *and* to conjoin propositions and never to conjoin noun phrases, verb phrases, or individual words (with one possible exception and two indeterminate instances where errors obscured what units H.M. was trying to conjoin).

#### 5.2.3. Use Frequency of *and versus* Other Ways of Conjoining Propositions

Why did H.M. overuse *and* but no other coordinating conjunction relative to the controls? And why did H.M. only use *and* to conjoin propositions rather than phrases or isolated words? Related to these questions is a third question: Does H.M. also overuse other means of conjoining propositionsrelative to the controls? To address this question, Study 3 examined how often H.M. conjoined propositions using correlative conjunctions* (either ... or*, *neither ... nor*, *both ... and*, and *not only ... but also)*; subordinating conjunctions (*after*, *before*, *unless*, *although*, *if*, *until*, *as*, *since*, *when*, *because*, *whereas*, and *while)*; and complementation structures (infinitive clauses, as in *He hopes* ***to leave early***; gerund clauses, as in *He enjoys **doing that***; *that* clauses, as in *She hinted **that** we should get the lead out*; and *who* clauses, as in *He knew **who** came*).

Together, mean use frequencies for correlative conjunctions, subordinating conjunctions, and propositional complementation did not differ reliably for H.M. (0.078 per word) *versus* the controls (0.078 per word*; SD* = 0.021), unlike propositional conjunction via *and*, which did differ reliably for H.M. (0.139 per word) *versus* the controls (0.102 per word; *SD* = 0.018). In short, relative to the controls, H.M. overused *and* but no other means of conjoining propositions.

### 5.3. Subsidiary Results: Troubles Accompanying H.M.’s Propositional Conjunctions

#### 5.3.1. Run-on Sentences: Trouble Linked With *and* But No Other Propositional Conjunction

Run-on sentences conjoin semantically unrelated themes or topics, a type of trouble reliably associated with H.M.’s use of *and*. Examples are (11), where *and*conjoins two semantically unrelated themes (*it is wrong for her to be **and** the way he’s dressed)*, and (12), where *and* conjoinsfour unrelated themes: pie was back here ***and*** *coffee is in there **and** this is boiled milk **and** this is not liquid.* There were no examples where H.M. produced run-on sentences using otherways of conjoining propositions and the controls never produced run-on sentences (a reliable 6.0 *SD* difference by convention).

(11). H.M.: “it’s wrong for her to be **and** he’s dressed just as this ...” (Run-on sentence; see the supplementary materials for H.M.’s complete utterance).
(12). H.M.: “Well this pie is—or the pie here was back here—**and** uh coffee is in there because heat a solid **and** this is only boiled milk say milk there **and** this is not liquid but only ice.” (Run-on sentence)

#### 5.3.2. Troubles Accompanying All Propositional Conjunctions

H.M.’s use of *and* shared three types of trouble with other propositional conjunctions: ungrammatical uses, inaccurate references, and non-sequiturs. However, propositional conjunctions of the controls exhibited none of these troubles (reliable 6.0 *SD* differences by convention).

##### 5.3.2.1. Ungrammatical Uses

Both omission- and commission-type misuses of *and* rendered H.M.’s sentences ungrammatical. In omission-type misuses such as (13) and (14), H.M. omitted one of the two or more entities that *and* must conjoin, thereby violating the TLC instruction to produce grammatical sentences.

(13). H.M.: “**And** he has to use his legs to climb.” (incomplete sentence)
(14). H.M.: “**And** that man is trying to tell that woman not to sit there because it’s wet paint.” (incomplete sentence)

Commission-type misuses of *and* rendered sentences ungrammatical by violating the “same-syntax rule” or “coordinative structure constraint.” Under the same-syntax rule, coordinating conjunctions must conjoin units in the same lexical or syntactic category, e.g., two main verbs, as in *I have seen **and** heard Wagner’s Tannhauser*; two noun phrases, as in *I went to the symphony **and** the opera*; or two propositions, as in *I want that **and** it’s available* (see, e.g., [63]). H.M. often violated this same-syntax rule by using *and* to conjoin different lexical or syntactic categories, thereby rendering his sentences ungrammatical, incoherent, and difficult to understand. For example, under one interpretation, H.M.’s *and* in (15) conjoins a verb phrase (*traveled on that bus*) with a proposition (*have it drive it off*), a violation of the same-syntax rule.

(15). H.M.: “Melanie tra … on that bus, the scrawny bus **and** have it drive it off … it, it drives it off.” (uncorrected misuse of *and*)

H.M. also produced ungrammatical sentences using other means of conjoining propositions, as in (16a–d), where he misused the subordinating conjunctions *because* and *if* without correction, and in(16e), where he produced the uncorrected error *some her* when conjoining a proposition with a complement.

(16a). H.M.: “**Because** it’s too hard to do it that way.” (Uncorrected misuse of subordinating conjunction in bold: incomplete sentence)
(16b). H.M.: “**Because** it’s wrong for her to be and he’s dressed just as this that he’s dressed.” (Uncorrected misuse of subordinating conjunction in bold: incomplete sentence)
(16c). H.M.: “And that man is trying to tell that woman not to sit there **because** it’s wet paint.” (Uncorrected misuse of subordinating conjunction in bold: ***because*** *it’s wet paint* substituted for ***because*** *the paint is wet*)
(16d). H.M.: “**If** they don’t use legs like he does … and his hands, they could fall.” (Uncorrected misuses in a subordinating conjunction in bold: omission of *their* in *their legs*, substitution of *his hands* for *their hands*)
(16e). H.M.: “I like some her … what she had.” (Uncorrected error *some her*, plus omission of *of* in the complement *of what she had* was)

##### 5.3.2.2. Inaccurate References

H.M.’s uses of *and* often falsely characterized a TLC picture, as in (17), where “**and** the same way as her” inaccurately describes a male and female customer in a clothing store as similarly dressed (whereas the male customer and male clerk are similarly dressed). To accurately describe the picture, H.M. should have said something like ***and*** *he’s dressed the same way as this man is*. Likewise in (18), H.M.’s “**and** he is just waiting to get waited on” inaccurately describes a man being waited on in a cafeteria: To accurately describe the picture, H.M. should have said something like ***and*** *he is just getting waited on*.

(17). H.M.: “Because it’s wrong for her to be **and** he’s dressed just as this that he’s dressed **and** the same way—as her.” (Inaccurate reference)
(18). H.M.: “He is getting some of this **and** it isn't pointed out here what it is **and** he is just waiting to get waited on.” (Inaccurate reference)

H.M. also produced inaccuracies using other propositional conjunctions, as in (19), where the subordinating conjunction *because* inaccurately describes the TLC picture because people are neither right nor wrong to be, contrary to the implication of H.M.’s “**because** it’s wrong for her to be.”

(19). H.M.: “**Because** it’s wrong for her to be.” (Inaccurate reference)

##### 5.3.2.3. Non-sequiturs

H.M.’s use of *and* often yielded non-sequiturs or logical contradictions, as in (20), where “***and*** to see what he’s using to pull himself up besides his hands” logically contradicts H.M.’s preceding proposition: “David wanted him to fall.” If the climber in the picture fell, David would be unable to see how he was pulling himself up.

(20). H.M.: “David wanted him to fall **and** to see what he’s using to pull himself up besides his hands.” (Non-sequitur involving use of ***and***; For expository reasons, we have corrected several irrelevant errors in (20): see the supplementary materials for H.M.’s uncorrected utterance).

H.M.’s subordinating conjunctions also yielded non-sequiturs, as in (21), where “**because** heat a solid” is logically unrelated to H.M.’s sentence topic, the location of coffee and pie in the picture.

(21). H.M.: “Well this pie is—or the pie here was back here—and uh coffee is in there **because** heat a solid.” (non-sequitur associated with *because*)

### 5.4. Discussion

Why was *and* the only way of conjoining propositions that H.M. overused relative to the controls? And why did H.M. overuse *and* even though other ways of conjoining propositions were less prone to “trouble” (run-on sentences)? To address these and other questions raised by the present results, we developed the compensation hypothesis discussed next.

#### 5.4.1. The Compensation Hypothesis

Under the compensation hypothesis, H.M. has difficulty forming coherent plans for producing novel (never-previously encoded) phrases and sentences, and to compensate for this difficulty on the TLC, H.M. generated familiar (previously encoded in immediate or long term memory) propositions via free association and used *and* to conjoin them into sentences. This proposition-level free association + *and* strategy complied with the TLC instruction to produce a single grammatical sentence (because propositions conjoined via *and* are grammatical under the same-syntax rule), but caused a “troublesome” side effect shared by no other way of conjoining propositions: run-on sentences consisting of unrelated propositions. Nonetheless, ungrammatical sentences, inaccurate references, non-sequiturs, and uncorrected misuses also accompanied these other ways of conjoining propositions because of H.M.’s difficulty in creating never-previously encoded sentences that are coherent and accurate.

This compensation hypothesis raises four basic questions: Does H.M. have difficulty forming coherent plans for producing novel sentences? Does H.M. produce reliably more free associations than memory-normal controls when creating novel sentences? Do H.M.’s uses of *and* on the TLC fit the standard definition of free association? And how did H.M.’s free association + *and* strategy benefit his TLC performance under the compensation hypothesis? As discussed next, evidence bearing on these and other questions indicates that the compensation hypothesis is sufficiently plausible to warrant further test.

#### 5.4.2. Does H.M. Have Difficulty Forming Coherent Plans for Novel Phrases and Sentences?

Forming coherent plans for producing novel phrases and sentences has been problematic for H.M. in a wide range of tasks; see, e.g., [3,5,8,9,10,64,65], and [2] (as analyzed in [6]).

#### 5.4.3. H.M.’s Propositional Conjunctions: Free Association as Classically Defined?

As classically defined (see [66]), words produced via free association are unrelated or inappropriate to the current situational or conceptual context but strongly related to thoughts, events, or concepts with preformed associations in memory. Consistent with this classical definition, H.M. often used *and* to conjoin concepts with preformed associations in memory but no obvious relation to the current conceptual or situational context, here, the instruction to use the target words to accurately describe a TLC picture. For example, preformed associations in semantic memory between *waiting* and *waited* in (18) almost certainly triggered H.M.’s inaccurate claim that the man *is waiting to get waited on* rather than *is being waited on*, as clearly indicated in the TLC picture. Similarly, preformed associations in memory between the concepts *heat*, *solids*, and *liquids* in (12) almost certainly triggered H.M.’s irrelevant non-sequitur “because heat a solid” in “coffee is in there because heat a solid **and**...”.

Also consistent with the classical definition of free association, irrelevant (or imagined) aspects of the pictures often triggered *and*-linked thoughts unrelated to the TLC goals, as in (11), where H.M. said “it is wrong for her to be **and** the way he’s dressed”, and in (12), where H.M. said “pie was back here **and** coffee is in there **and** this is boiled milk **and** this is not liquid”.

#### 5.4.4. How Did H.M. Benefit from His Free Association + *and* Strategy?

Under the compensation hypothesis, H.M. used *and* to conjoin two or more propositions retrieved via free association, thereby compensating for his difficulties in constructing coherent sentence-level plans. This proposition-level free association + *and* strategy obviated the need to construct an overall sentence plan because any two propositions conjoined via *and* yield a sentence that satisfies the same-syntax rule and the TLC instruction to produce a single grammatical sentence. For example, H.M.’s conjoined propositions in “pie was back here **and** coffee is in there **and** this is boiled milk **and** this is not liquid” yield a single sentence that is grammatical but incoherent and run-on.

#### 5.4.5. Why Did H.M. Prefer to Conjoin Propositions via *and*?

Using *and* to conjoin propositions involves simpler, more general, and less constrained processes than other ways of conjoining propositions. Only one relation between the conjoined units (the same-syntax concatenation rule) must be computed when using *and*, whereas two additional and more complex relations must be computed when using the subordinating conjunctions *although*, *after*, and *because*: *Although* requires computation of concatenation, subordination, and contrary relations; *after* requires computation of concatenation, subordination, and temporal relations; and *because* requires computation of concatenation, subordination, and causal relations. To compensate for his deficits in forming grammatical plans for novel sentences, H.M. therefore preferred *and* as the easiest way to conjoin propositions retrieved via free association under the compensation hypothesis.

#### 5.4.6. Why Was H.M.’s *and* More “Troublesome” Than Other Propositional Conjunctions?

H.M.’s added trouble with *and* only showed up as run-on sentences, not other types of misuses, and directly reflected his free association + *and* strategy under the compensation hypothesis. However, the remaining “troubles” associated with *any* way of conjoining propositions (ungrammatical sentences, inaccurate references, non-sequiturs, and uncorrected misuses) reflect a more general cause under the compensation hypothesis: H.M.’s inability to form coherent plans or internal representations for novel phrases and propositions.

## 6. Summary, Conclusions and Caveats

Use frequency analyses in Study 1 provided our first major result: that H.M. could retrieve (at least) 21 lexical categories and one syntactic category (noun phrases) with no lower relative frequency than matched memory-normal controls on the TLC. This finding contrasts with the underuse of specific lexical and syntactic categories that characterizes category-specific aphasia, and suggests that H.M.’s category-specific mechanisms for retrieving words in phrases and phrases in sentences are intact.

Also consistent with intact brain mechanisms for retrieving already encoded phrases, H.M. has produced many familiar phrases without errors in conversational speech since his lesion. Examples are the six multi-word noun phrases in H.M.’s brief paragraph in (1): “a lay teacher”, “the kids”, “the nuns”, “the grade”, “the next grade”, “young kids”, and “in a way”. H.M. almost certainly encoded all six phrases prior to his lesion, and his error-free use of familiar phrases was probably one of the reasons why researchers interacting informally with H.M. since his lesion (mistakenly) assumed that his language skills were completely intact (see [3,6] for additional reasons).

The simplest explanation of Study 1 use-frequency results is that (a) frontal areas contain the activating-mechanisms for retrieving already-encoded words, phrases, and propositions, and (b) retrieval mechanisms in H.M.’s frontal cortex are intact. Amnesics with compound frontal and hippocampal damage such as Clive Wearing reinforce and extend this account. Consistent with their hippocampal damage, Clive and H.M. cannot form new episodic memories (except via massive repetition; see [54]). However, using his intact frontal cortex, H.M. can retrieve episodic memories encoded before his lesion, whereas due to his frontal damage, Clive cannot ([67], pp. 187-213).

Studies 1 and 2 provided our second major result: H.M.’s reliable overuse of proper names in speech and writing relative to memory-normal controls. In a follow-up study, MacKay *et al.* [54] examined H.M.’s use of proper names in detail and using the compensation hypothesis developed in Study 3, concluded that H.M. overused proper names to compensate for his inability to encode structures with the same function as proper names.

The compensation hypothesis in Study 3 was developed to explain H.M.’s reliable overuse of the coordinating conjunction *and* relative to memory-normal controls in Study 1. Under this hypothesis, H.M. overused *and* for three reasons: (a) to compensate for his inability to construct sentence-level plans that were novel, accurate, and grammatical [3,5,6,9,10,11,65,68]; (b) to conjoin familiar propositions into multi-proposition sentences; and (c) to satisfy the instruction to describe TLC pictures using a single grammatical sentence.

All three factors together contributed to a proposition-level strategy that fit the classical definition of free association but was more complex than word-level free associations observed to date (see [66]). Using this proposition-level free association strategy, H.M. retrieved familiar propositions via free association and conjoined them via *and*, the least constrained way of conjoining one or more propositions to form a grammatical sentence. This strategy obviated the need to form a novel sentence plan and satisfied the TLC instruction to produce a single grammatical sentence, but carried a negative consequence seen with none of the other propositional conjunctions that H.M. used: run-on sentences (see also the negative consequences of H.M.’s proper name compensation strategy in MacKay *et al*. [54]).

Several caveats are in order regarding the present results and conclusions. One is that H.M.’s normal use-frequency profile for noun phrases and (at least) 18 lexical categories in Study 1 does not imply that his language skills are “relatively intact” or “unimpaired”: H.M.’s ungrammatical uses, inaccurate references, run-on sentences, and non-sequiturs in Study 3 indicate that his mechanisms for *encoding* new phrases, propositions, and sentences are impaired (see also [54]).

Another caveat is that the compensation hypothesis described the observations in Study 3, but did not predict them. New observations are needed to test the compensation hypothesis (a process undertaken in [54]).

As a final caveat, the present use frequency results are specific to H.M. rather than to amnesia in general: Amnesics with different types of brain damage can be expected to compensate in different ways (for additional caveats, see [54]). For example, it is unsurprising that the amnesic patients in Almor, Kempler, MacDonald, Andersen and Tyler [69] used reliably more pronouns than memory-normal controls, whereas H.M. used pronouns with the same relative frequency as memory-normal controls in Studies 1–2. The Almor *et al.* [69] amnesics were compensating for diffuse cortical damage linked to Alzheimer’s Disease, whereas H.M. had virtually no cortical damage and was compensating for hippocampal region damage. Additional case studies therefore seem warranted to explore the parameters and range of category-specific compensation in amnesics with different types of brain damage. As Ramachandran ([70], p. xi) notes, careful study of single cases has in the past proved instrumental in discovering most, and perhaps all, of the syndromes in neurology.

## Supplementary Files

Supplementary File 1 Supplementary Materials (PDF, 142 KB)

## References

[1] Milner B., Corkin S., Teuber H.-L. (1968). Further analysis of the hippocampal amnesic syndrome: 14-year follow-up study of H.M. Neuropsychologia.

[2] Marslen-Wilson W. Biographical interviews with H.M. 1970. Unpublished transcript. http://www.mackay.bol.ucla.edu.

[3] MacKay D.G., James L.E., Hadley C.B., Fogler K.A. (2011). Speech errors of amnesic H.M.: Unlike everyday slips-of-the-tongue. Cortex.

[4] MacKay D.G., James L.E. (2002). Aging, retrograde amnesia, and the binding problem for phonology and orthography: A longitudinal study of “hippocampal amnesic” H.M. Aging Neuropsychol. Cogn..

[5] MacKay D.G., Hadley C.B. (2009). Supra-normal age-linked retrograde amnesia: Lessons from an older amnesic (H.M.). Hippocampus.

[6] MacKay D.G., Burke D.M., Stewart R. (1998). H.M.’s language production deficits: Implications for relations between memory, semantic binding, and the hippocampal system. J. Mem. Lang..

[7] Corkin S. (1984). Lasting consequences of bilateral medial temporal lobectomy: Clinical course and experimental findings in H.M. Semin. Neurol..

[8] MacKay D.G., James L.E. (2001). The binding problem for syntax, semantics, and prosody: H.M.’s selective sentence-reading deficits under the theoretical-syndrome approach. Lang. Cogn. Process..

[9] MacKay D.G., James L.E., Hadley C.B. (2008). Amnesic H.M.’s performance on the language competence test: Parallel deficits in memory and sentence production. J. Clin. Exp. Neuropsychol..

[10] MacKay D.G., James L.E., Taylor J., Marian D.E. (2007). Amnesic H.M. exhibits parallel deficits and sparing in language and memory: Systems *versus* binding theory accounts. Lang. Cogn. Process..

[11] MacKay D.G., Stewart R., Burke D.M. (1998). H.M. revisited: Relations between language comprehension, memory, and the hippocampal system. J. Cogn. Neurosci..

[12] Corkin S., Amaral D.G., González R.G., Johnson K.A., Hyman B.T. (1997). H. M.’s medial temporal lobe lesion: Findings from magnetic resonance imaging. J. Neurosci..

[13] Lashley K.S., Jeffress L.A. (1951). The problem of serial order in behavior. Cerebral Mechanisms in Behavior.

[14] Skotko B.G., Rubin D.C., Tupler L.A. (2008). H.M.’s personal crossword puzzles: Understanding memory and language. Memory.

[15] Bates E., Chen S., Tzeng O., Li P., Opie M. (1991). The noun-verb problem in Chinese aphasia. Brain Lang..

[16] Berndt R.S., Haendiges A.N., Burton M.W., Mitchum C.C. (2002). Grammatical class and imageability in aphasic word production: their effects are independent. J. Neurolinguistics.

[17] Caramazza A., Hillis A.E., Leek E.C., Miozzo M., Hirschfeld L.A., Gelman S.A. (1994). The organization of lexical knowledge in the brain: Evidence from category- and modality-specific deficits. Mapping the Mind.

[18] De Renzi E., Di Pellegrino G. (1995). Sparing of verbs and preserved but ineffectual reading in a patient with impaired word production. Cortex.

[19] Jonkers R., Bastiaanse R. (1998). How selective are selective word class deficits? Two case studies of action and object naming. Aphasiology.

[20] Job R., Miozzo M., Sartori G. (1993). On the existence of category-specific impairments. A reply to Parkin and Stewart. Q. J. Exp. Psychol. A.

[21] Kemmerer D., Tranel D. (2003). A double dissociation between the meanings of action verbs and locative prepositions. Neurocase.

[22] Luzzatti C., Raggi R., Zonca G., Pistarini C., Contardi A., Pinna G.-D. (2001). On the nature of the selective impairment of verb and noun retrieval: The role of word frequency and of imageability. Cortex.

[23] McCarthy R., Warrington E.K. (1985). Category specificity in an agrammatic patient: The relative impairment of verb retrieval and comprehension. Neuropsychologia.

[24] Miceli G., Silveri M.C., Nocentini I., Caramazza A. (1988). Patterns of dissociation in comprehension and production of nouns and verbs. Aphasiology.

[25] Miceli G., Silveri M.C., Villa G., Caramazza A. (1984). On the basis for the agrammatic’s difficulty in producing main verbs. Cortex.

[26] Mondini S., Luzzatti C., Semenza C., Calza A. (1997). Prepositional compounds are sensitive to agrammatism: Consequences for models of lexical retrieval. Brain Lang..

[27] Rapp B., Caramazza A., Sarno M.T. (1998). Lexical deficits. Acquired Aphasia.

[28] Silveri M.C., Di Betta A.M. (1997). Noun-verb dissociations in brain-damaged patients: Further evidence. Neurocase.

[29] Sartori G., Miozzo M., Job R. (1993). Category-specific naming impairments? Yes. Q. J. Exp. Psychol. A.

[30] Tabossi P., Collina S., Caporali A., Pizzioli F., Basso A. (2010). Speaking of events: The case of C.M. Cogn. Neuropsychol..

[31] Tyler L.K., Bright P., Fletcher P., Stamatakis E.A. (2004). Neural processing of nouns and verbs: The role of inflectional morphology. Neuropsychologia.

[32] Tyler L.K., Cobb H. (1987). Processing bound grammatical morphemes in context: The case of an aphasic patient. Lang. Cogn. Process..

[33] Zingeser L.B., Berndt R.S. (1990). Retrieval of nouns and verbs in agrammatism and anomia. Brain Lang..

[34] Goodglass H., Kaplan E. (1983). The Assessment of Aphasia and Related Disorders.

[35] Ulatowska H.K., Chapman S.B., Bloom R.L., Obler L.K., de Santi S., Ehrlich J.S. (1994). Discourse macrostructure in aphasia. Discourse Analysis and Applications: Studies in Adult Clinical Populations.

[36] Goodglass H. (2001). The Assessment of Aphasia and Related Disorders.

[37] Albert M.L., Goodglass H., Helm N.A., Rubens A.B., Alexander M.P. (1981). Clinical Aspects of Dysphasia.

[38] Blumstein S. (1973). A Phonological Investigation of Aphasic Speech.

[39] Nicolas L., Harryman E., Kreshek J. (1978). Terminology of Speech Disorders.

[40] Saffran E.M., Schwartz M.F., Marin O.S.M. (1980). The word order problem in agrammatism: II. Production. Brain Lang..

[41] MacKay D.G. (1987). The Organization of Perception and Action: A Theory for Language and Other Cognitive Skills.

[42] Burke D.M., Shafto M.A., Craik F.I.M., Salthouse T.A. (2004). Language and Aging. The Handbook of Aging and Cognition.

[43] Garnham A., Shillcock R.C., Brown G.D.A., Mill A.I.D., Cutler A., Cutler A. (1982). Slips of the tongue in the London-Lund corpus of spontaneous conversation. Slips of the Tongue and Language Production.

[44] Fromkin V.A. (1973). Appendix: A sample of speech errors. Speech Errors as Linguistic Evidence.

[45] MacKay D.G. (1970). Spoonerisms: The structure of errors in the serial order of speech. Neuropsychologia.

[46] Dell G.S. (1986). A spreading-activation theory of retrieval in sentence production. Psychol. Rev..

[B47-brainsci-03-00262] 47.H.M. died at age 82 in 2008. Our use of present tense refers not to the present publication date but to H.M. when tested from ages 44 to 71 in the present research

[48] Wiig E.H., Secord W. (1988). Test of Language Competence: Expanded Edition.

[49] Scoville W.B., Milner B. (1957). Loss of recent memory after bilateral hippocampal lesions. J. Neurol. Neurosurg. Psychiatry.

[50] MacKay D.G., Johnson L.W. (2012). Error detection, error correction, and medial temporal lobe amnesia: H.M. and mirror neuron theory. Neuropsychologia.

[51] Salat D.H., van der Kouwe A.J.W., Tuch D.S., Quinn B.T., Fischl B., Dale A.M., Corkin S. (2006). Neuroimaging H.M.: A 10-year follow-up examination. Hippocampus.

[52] Loftus M., Knight R.T., Amaral D.G. (2000). An analysis of atrophy in the medial mammillary nucleus following hippocampal and fornix lesions in humans and nonhuman primates. Exp. Neurol..

[53] Wilkinson A., Davies I. (1978). The influence of age and dementia on the neurone population of the mammillary bodies. Age Ageing.

[54] MacKay D.G., Johnson L.W., Hadley C.B. (2012). Compensating for language deficits in amnesia II: H.M.’s spared and impaired encoding categories. Brain Sci..

[55] Chomsky N. (1957). Syntactic Structures.

[56] Dryer M.A. (2007). Noun phrase structure. Language Typology and Syntactic Description: Complex Constructions.

[57] Givón T. (2001). Syntax: An Introduction.

[58] (2000). The American Heritage Dictionary of the English Language.

[59] Sagar H.J., Cohen N.J., Corkin S., Growdon J. (1985). Dissociations among processes in remote memory. Ann. N. Y. Acad. Sci..

[60] Burke D.M., MacKay D.G., Worthley J.S., Wade E. (1991). On the tip of the tongue: What causes word finding failures in young and older adults?. J. Mem. Lang..

[61] Cohen G., Burke D.M. (1993). Memory for proper names: A review. Memory.

[62] Semenza C. (2009). The neuropsychology of proper names. Mind Lang..

[63] Bach E. (1983). On the relationship between word-grammar and phrase-grammar. Nat. Lang. Linguist. Theory.

[64] MacKay D.G., Bechtel W., Graham G. (1998). Stage theories refuted. A Companion to Cognitive Science.

[65] MacKay D.G., James L.E. (2009). Visual cognition in amnesic H.M.: Selective deficits on the What’s-Wrong-Here and Hidden-Figure tasks. J. Clin. Exp. Neuropsychol..

[66] Cohen E.D. (1975). C. G. Jung and the Scientific Attitude.

[67] Sacks O. (2007). Musicophilia: Tales of Music and the Brain.

[68] James L.E., MacKay D.G. (2001). H.M., word knowledge, and aging: Support for a new theory of long-term retrograde amnesia. Psychol. Sci..

[69] Almor A., Kempler D., MacDonald M.C., Andersen E.S., Tyler L.K. (1999). Why do Alzheimer patients have difficulty with pronouns? Working memory, semantics, and reference in comprehension and production in Alzheimer’s disease. Brain Lang..

[70] Ramachandran V.S. (2004). A Brief Tour of Human Consciousness.

